# A New Approach to Body Donation for Medical Education: The Silent Mentor Programme

**DOI:** 10.5704/MOJ.1807.015

**Published:** 2018-07

**Authors:** A Saw

**Affiliations:** Department of Orthopaedic Surgery, University of Malaya, Kuala Lumpur, Malaysia

**Keywords:** anatomy, empathy, compassion, medical student, cadaver, cadaveric dissection

## Abstract

Cadaveric dissection is an integral component of medical education. There had been concerns about negative impact on medical students exposed to deceased donors before their clinical years, but most studies reported overall positive outcome following this form of teaching. Due to reducing number of body donations in most parts of the world, many institutions are adopting alternative models especially for the teaching of gross anatomy. A new body donation programme that incorporate humanistic values in the procurement process was initiated by Tsu Chi University of Taiwan in 1996. Early observations following teaching with the so-called “silent mentors” noted less negative emotional impact on the students. With increasing number of body donation following the initiation of the silent mentor programme as reported in some regions, we will be able to continue the time-honoured cadaveric dissection for anatomy teaching, at the same time promoting humanistic values on junior doctors.

## Historical Perspective

The structure and composition of the human body have been subject of interest among those interested to better understanding of disease and looking for its cure. Dissection of bodies of executed criminals has been practised since the period of the ancient Greek civilisation^1^. The word ‘anatomy’ was derived from the Greek and Latin words “ana” and “tomia” meaning “to cut up”. With the development of medical education, the study of gross anatomy became one of the basic components that contributed towards the understanding of physiological and pathological processes of the human body.

**Fig. 1:**
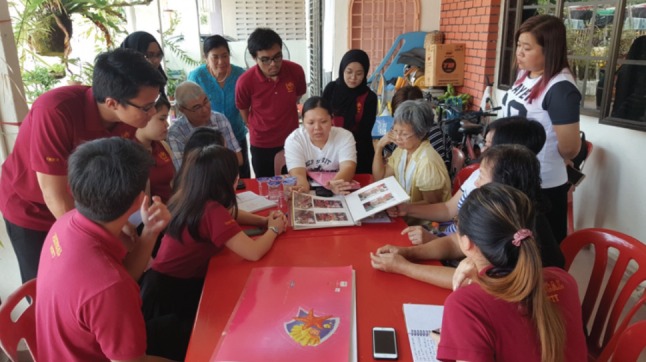
A group of medical students from a few local universities visited the family of their mentor to get to know his / her family background.

During the 19th century, medical students in Europe and North America studied the gross anatomy of the human body from the dissection of unclaimed corpses or donated bodies from families who were too poor to arrange for proper burial. Such was the demand for the bodies that some even resorted to stealing bodies from fresh graves to offer to medical institutions or individuals for profit^2^. Until the middle of the 20th century, cadaveric dissection remained the core syllabus of medical education where medical students would spend one or more years dissecting preserved human bodies in the dissection halls (Fig. 1) At the end of the 20th century, the improving standards of living and increasing life expectancy resulted in diminishing sources of fresh human bodies available for medical education, especially among the more developed countries. Other countries had also restricted or prohibited the export of human body or body parts for medical education. Alternative learning modules are being developed, with new preservation methods to prolong the usage of available body specimens.

## Learning Gross Anatomy

The study of gross anatomy is an important component of medical education for doctors, dentists and pharmacists. Few will disagree that cadaveric dissection is the most effective way to learn about gross anatomy and physiology. There has been concern about the negative aspects of exposure of medical students to dead bodies^3^-^5^. There are various coping methods and mechanisms towards these negative experiences. Most studies concluded that medical students who participated in cadaveric dissection would eventually overcome their stress and anxiety, and benefit from the exposure^6^-^9^. However, it has been shown that some of these coping mechanisms, including detachment and indifference to the cadaver and its dissection, may be reinforced during the course, and eventually transform into an ingrained negative attitude after the student graduates as a doctor^10^.

With decreasing donated bodies in many countries, alternative methods have been developed to help medical students learn gross anatomy. Instead of assigning students to a cadaver and allowing them to explore the structures by dissection, prosection is carried out in many centres where the cadaver will be dissected by experienced anatomists in demonstrations to the students^11^ This method allows the tissue or organ specimens to be used for repeated sessions of teaching over many years. Plastination is another method that allows prolonged preservation of human body model for teaching where body fluids are replaced by durable polymers that preserve the properties of the original tissue^11^.

Anatomical models of the human body, fabricated from different types of synthetic materials, are also available for teaching and learning. With the advancement of computer technology, many programmes can provide three-dimensional images that help medical students learn and appreciate the human anatomy. However, most academicians and clinicians would attest that learning through dissection of cadaver remains the most effective way for medical students to learn gross anatomy^12^,^13^.

## Surgical Simulation

The training of basic surgical skills is another important component of medical education. Traditionally, students learn how to perform basic bed-side procedures by observing their seniors or the physicians performing the procedures. The procedures include, among others, the setting of lines, the drainage of skin abscesses, and the suturing of skin lacerations. For more complex procedures like endotracheal intubation, or drainage of thoracic or abdominal cavities, the students may need to assist in a few cases, then to perform under supervision, before being allowed to proceed independently. The main problem is that opportunities for these procedures may be few and not be present all the time. The medical simulation mannequin or “virtual patient” was initially developed for the training of resuscitation procedures in emergency medicine. This is now widely used for undergraduate and various subspecialty medical training. Invasive or non-invasive procedures can be performed on the mannequin repeatedly. However, for more delicate surgical procedures, these models will not provide an experience that simulates performing on a live patient.

**Fig. 2:**
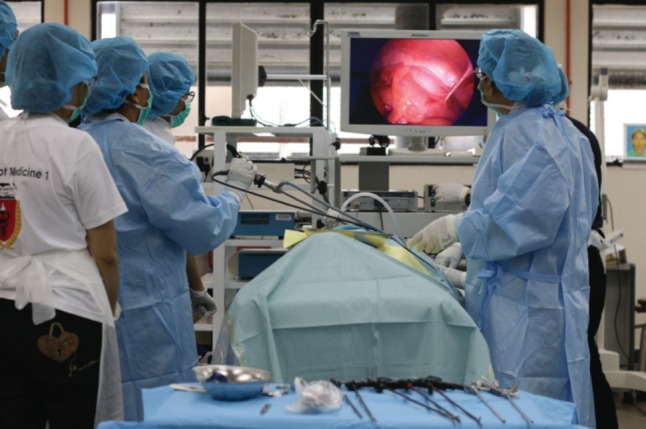
Laparoscopic surgery is one of the most common procedure where surgeons can benefit from surgical simulation using cadavers.

Surgical simulation using cadavers are considered one of the most effective ways for a doctor to acquire practical skills before embarking on performing procedures independently. Some procedures like arthroscopy and microscopic surgery require high-level coordination between the brain and the hand, and practising on cadavers is one of the few methods that can be used to achieve the training objective (Fig. 2). However, the limited availability of cadaver severely restricts this mode of training in most countries.

## Modern Medical Education

Modern medical education incorporates the three core aspects of knowledge, skill and humanity. Traditionally, they are taught in two stages, the preclinical and clinical phases, by academic staff who are mainly familiar with their own fields of specialty. Preclinical academic staff including the anatomists and the physiologists, hardly see sick patients, while practising physicians and surgeons rarely step into the preclinical lecture halls. Under the stress of examination, many medical students in their clinical years, will only focus on their acquisition of the necessary medical knowledge and on completing their log books to enable them to qualify for their final evaluation.

The materials taught during the preclinical years appear to many medical students obsolete and irrelevant to their future professional development. All these may contribute towards the general decline in the humanistic component of medical training. Deterioration in doctor-patient relationship has become a major problem in many parts of the world in the training systems^14^. The decline and the deterioration may be due to the higher expectation from patients and an increasing tendency towards medicolegal litigation. The change in the practice and attitude among new graduates may also be an important factor. Clinical decisions are often influenced by financial returns and medical policies influenced by commercial interests. Patients may no longer the main focus of the profession.

With an increasing number of medical schools in both developed and developing countries, the quality of newly trained doctors has been a grave concern. Many medical schools have introduced an integrated medical curriculum that combined clinical and non-clinical aspects of training throughout the program^15^. A more continuous form of evaluation has also been adopted because this may be a more effective method to evaluate the humanistic component of medical education. The level of knowledge can be assessed through many types of the conventional evaluation system. It is more difficult to assess the proficiency in performing clinical or surgical procedures and the attitude towards the disease, the patients and the society. Few education models have been shown to be effective in instilling the humanistic quality of compassion and empathy in the students. This is one of the most important qualities that patients expect from a good doctor. Furthermore, in the most education system, there are limited contacts between the students with the community and the healthy individuals. Despite the fact that there is an increasing interest in a healthy life style and disease prevention, the main approach towards medicine education is still based on the sick and on those seeking treatment.

## The Silent Mentor Programme

Voluntary body donation is still practised in many developing countries because there is no shortage of donors^16^. In more developed countries, dissection of cadavers has stopped due to a lack of body donors. In 1996, the Tzu Chi University initiated a novel body donation programme with voluntary donors^17^. Through home visits, the medical students get to know the personal and the family background of the donor, as well as the social and medical history. They will prepare a presentation of their assigned donors, whom they will address as their teacher or mentor, during the whole learning process. This is in contrast to the common practice in many medical schools where most students do not know the name or the family background of the donors^18^. In the silent mentor programme, there will be an initiation ceremony, where family members of all the donors meet the students just before the training session. Chiou *et al* have shown that the attitude towards death and the level of negative emotions were reduced after the students attended an initiation ceremony of the silent mentor program in the presence of the donor family^19^ At the end of the training session, there would be an appreciation and a sending off ceremony where all the students and the academic staff who would have participated in the training sessions would be invited to express their gratitude to the mentors and their family members.

**Fig. 3:**
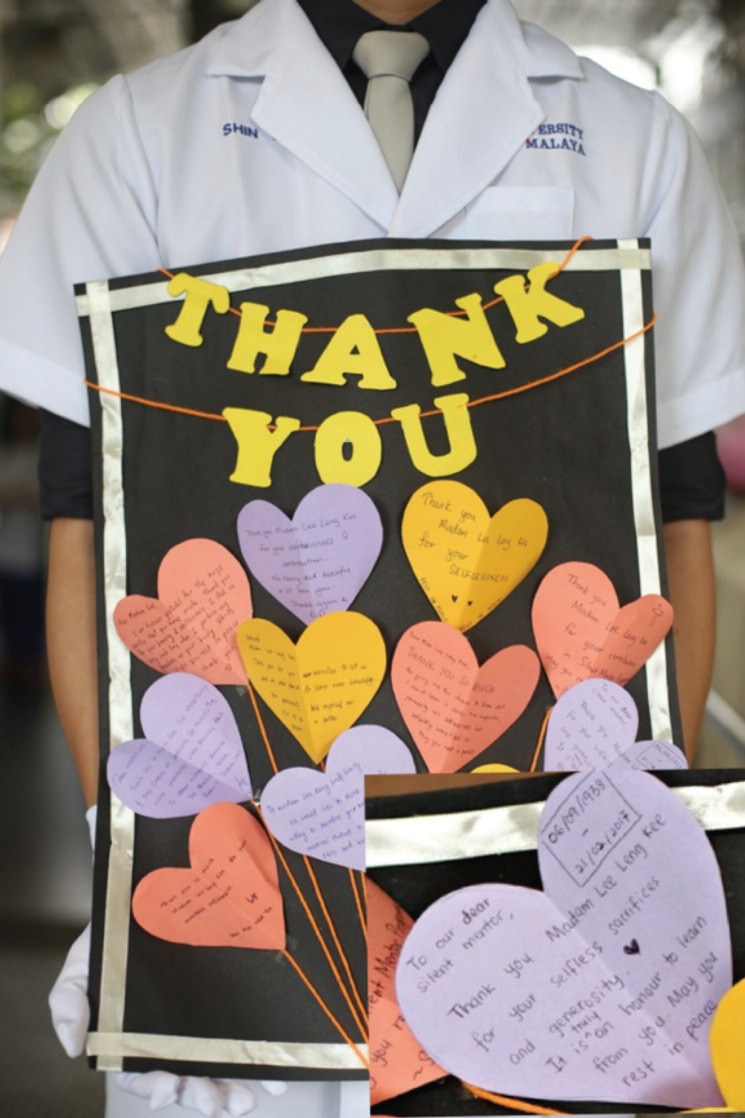
Souvenirs from medical students dedicated to the mentors on the sending off ceremony.

In 2012, University of Malaya collaborated with the Tzu Chi University and started the first silent mentor programme outside Taiwan. Over the following years, more than 80 silent mentors have contributed to the training of several hundreds of medical students from various teaching institutions in Malaysia and its neighbouring countries. Based on the feedback from the medical students and medical doctors who participated in the programme, the experience has been very positive, with a better understanding of anatomical structures, the acquisition of clinical and surgical skills, and an appreciation of the donors and the other potential donors who have pledged their bodies to the programme. Many of participants admitted that they were touched by the fact that people who they had not known or met, were willing to donate their body for their improvement, without expecting anything in return (Fig. 3). The long-term influence of establishing a close association between the students and the donor and the donor family cannot be predicted. Regarding the professional development of the students, this will be a positive move towards fostering a sense of compassion and empathy in them. The evidence supporting these observations is still lacking, and qualitative and quantitative evaluations, especially on the non-clinical aspects of the silent mentor body donation programme, would be valuable and hopefully forthcoming.

The public acceptance of the silent mentor programme has also been encouraging. The number of pledgers for body donation has been increasing over the years. More recently, some teaching institutions in Singapore, Hong Kong and Myanmar have started body donation programmes following the model of the the silent mentor programme. The The National University of Singapore has even revived its cadaveric dissection programme for undergraduate training, which had been stopped due to the limited supply of cadavers^20^.

## Future Development

Medical education has come to a cross-road where there is increased stress in acquiring knowledge with the lack of good modules for the teaching of humanistic values of this noble profession. After the formal medical education, junior doctors are exposed to the commercial influence and the pressure for better financial return. With increasing medico-legal litigation, many doctors resort to the practice of defensive medicine. In the long run, the patients will be the losers. Training modules that promote a sense of compassion and empathy among medical students and junior doctors so that they will respect their patients and continue to uphold the noble spirit of our esteemed profession are important. The various silent mentor programmes have the potential to contribute towards this objective.

The more objective evaluation may show a positive outcome from the centres with the Silent Mentor Programme or the like, with improving public awareness and acceptance . Medical education, in general, can then be further improved to produce not only knowledgeable and skilful doctors, but also those who are compassionate and empathetic towards patients and the society as a whole.

## Acknowledgement

I would like to thank Gracie Ong for her assistance in the preparation of this article.
